# Supplementary material for: From sequence to enzyme mechanism using multi-label machine learning
Source: BMC Bioinformatics. 2014 May 19;15:150. doi: 10.1186/1471-2105-15-150 (PMC4229970; doi:10.1186/1471-2105-15-150)
Supplement: Additional file 2 — Java code of ml2db. Additional file ml2db_code.tar.gz contains the Java source code to run the multi-label machine learning experiments and save the results to database. The code’s Javadoc is included. [file 1471-2105-15-150-S2.zip › additional file 2/ml2db/ml2db/doc/mulan/examples/package-summary.html]

mulan.examples


JavaScript is disabled on your browser.


- Overview
- Package
- Class
- Use
- Tree
- Deprecated
- Index
- Help

- Prev Package
- Next Package

- Frames
- No Frames

- All Classes

# Package mulan.examples

- Class Summary

  | Class | Description |
  | --- | --- |
  |  |  |
  | --- | --- |
  | CrossValidationExperiment | Class demonstrating a simple cross-validation experiment |
  | DimensionalityReductionTest | Demonstrates the attribute selection capabilities of Mulan |
  | EstimationOfStatistics | Class that calculates multi-label stastistics for a multi-label dataset |
  | GettingPredictionsOnUnlabeledData | This examples shows how you can retrieve the predictions of a model on unlabeled data. |
  | StoringAndLoadingModels | This example shows how you can store a learned model and load a stored model. |
  | SubsetLearnerExamples | A main class with examples for SubsetLearner, GreedyLabelClustering and EnsembleOfSubsetLearners methods usage. |
  | TrainTestExperiment | Class demonstrating a simple train/test evaluation experiment |

- Overview
- Package
- Class
- Use
- Tree
- Deprecated
- Index
- Help

- Prev Package
- Next Package

- Frames
- No Frames

- All Classes
